# Supplementary material for: Integrating Social Care into Healthcare: A Review on Applying the Social Determinants of Health in Clinical Settings
Source: Int J Environ Res Public Health. 2023 Oct 2;20(19):6873. doi: 10.3390/ijerph20196873 (PMC10573056; doi:10.3390/ijerph20196873)
Supplement: Supplementary file 1 [file ijerph-20-06873-s001.zip › ijerph-2568306-supplementary.pdf]

Supplementary Materials

Integrating Social Care into Health Care: Applying the Social Determinants of Health in Clinical Settings

M. Lelinneth B. Novilla, Michael C. Goates, Tyler Leffler, Nathan Kenneth B. Novilla, Chung-Yuan Wu, Alexa Dall, Cole Hansen

Table S1. Integrating SDH in the Clinical Setting by Study Design, Method of Integration, and Type of Health Care Facility Based on a Literature Review.

Author, Year Published, Reference Number	Study Design	Method of SDH Integration	Type of Healthcare Facility & Geographic Location
	Summary: Integrating SDH in clinical settings involved the following study designs:<br>• 19 quantitative studies;<br>• 9 qualitative studies:<br>• 5 mixed methods analyses;<br>• 2 systematic reviews, and<br>• 9 theoretical papers that included policy and literature reviews, commentaries, and opinions	Summary: Integrating SDH in clinical settings commonly involved using SDH screening tools during patient admission/check-in to collect patient-level SDH data to link patients to community resources and services. There was little information from the literature on how patient-level SDH data were being used across the care continuum and/or in addressing the underlying long-term social drivers of health inequities at the community level.	Summary: Integrating SDH in clinical settings frequently involved primary care and pediatric clinics, community health centers, and safety net hospitals. Most SDH integration mentioned in the literature occurred in urban versus rural communities.
Woolf et al. 2017 [18]	Commentary	Suggested use of SDH screening tool during check-in/admission (no specific tool mentioned); adding social workers to clinical staff; training and reimbursing physicians in addressing patients' social needs	Medical offices and clinics providing preventive and therapeutic health care services<br>Urban and rural communities
Parekh et al. 2022 [26]	Systematic Review	Use of systematic review with focus on "persistent geographic disparities in CVD health," i.e., food insecurity and housing instability as determinants of CVD outcomes; review cited inconsistent measures of housing instability on hospital/medical records (ICD codes), self-rated surveys, or face-to-face interviews; article listed 11 databases and surveys on food insecurity and housing instability; suggested use of national survey data to assess food insecurity: **NHIS, NHANES** (10-item food insecurity questions), **BRFSS** (1-3-item food insecurity questions); cited use of the **2-question food insecurity tool** by ProMedica, a health system in Ohio and Michigan, to screen hospitalized patients for food insecurity. This resulted in ProMedica opening two "food pharmacies" where patients were referred to by primary care physicians and counseled by dietitians	Nationwide medical offices, primary care clinics for obesity and diabetes, specialty clinics, hospitals, community health centers
Mahmood et al. 2023 [28]	Quantitative Study (Binary Logistic Regression)	Use of 2014 and 2016 waves of Health and Retirement Study and 2013 Health Care and Nutrition Study to determine association between food security and breast cancer screening (propensity score weighting used to balance observed confounders between food-secure and food-insecure women, and binary logistic regression to investigate population-level estimates for the association between food security and breast cancer screening)	Research team<br>University of Memphis School of Public Health
Franz et al. 2022 [47]	Quantitative Study	Use of county-level health and economic data to compare SDH characteristics of communities around non-profit children's hospitals and to categorize the SDH strategies in their IRS-mandated community benefit work	168 non-profit children's hospitals<br>National sample
DeVetter et al. 2022 [48]	Quantitative Study (Descriptive Analysis)	Use of search pattern analysis for the AAFP **Neighborhood Navigator (NN)** tool's Aunt Bertha/Find Help (AB/FH) referral platform in	Primary care clinics

		addressing severe social needs; tool was used by physicians and patients	
Synovec and Aceituno 2020 [80]	Policy and Literature Review	Use of policy and literature review to identify major SDH issues among patients with housing instability, including refugees, seen in an occupational therapy practice; discussed how SDH issues affected patients' occupational performance and ability to manage health conditions	Occupational Therapy clinic<br>Baltimore, MD, urban area
Millender et al. 2022 [49]	Quantitative Study (Retrospective Exploratory Analysis)	Use of **PHQ-9** for depression<br>No specific SDH screening tool mentioned	NLCHCs<br>South Florida, urban communities
Franz et al. 2019 [50]	Quantitative Study (Logistic Regression)	Use of dataset on hospital-led interventions to address opioid abuse in 20% randomly-selected non-profit hospitals to determine number of strategies adopted and whether such strategies were new, existing, or primarily partnerships with community-based organizations; dataset was based on publicly-available ACA-mandated CHNAs; no specific SDH screening tool mentioned.	US non-profit hospitals<br>National sample
Begun et al. 2018 [65]	Qualitative Study (Likert Scale)	Use of a 5-point impact scale created to determine the potential impact of 317 hospital-level community health activities on population health and equity as reported in CHNAs; 50% of reported community activities were classified as SDH ("community health and connectedness" and "healthy lifestyles and wellness"), but only 5% addressed structural causes of health equity; no specific SDH screening tool mentioned	23 health care organizations<br>Minneapolis & St. Paul, Minnesota<br>Metropolitan area
Brennan et al. 2022 [66]	Qualitative Study	Use of PDSA, monthly chart reviews of well-child visits, and collaborative meetings on the completion of SDH screenings; discussion of screening results with families, and referrals for positive screens; developed composite measures of performance from chart reviews; no specific SDH screening tool mentioned.	10 pediatric practices<br>Mid-central Indiana
Denny et al. 2019 [67]	Qualitative Study (Pilot Quality Improvement Study)	Use of the following:<br>• Standardized and age-appropriate screening hybrid tool during well-child visits that incorporated **SEEK** tool to identify common SDH risks among children aged 0-1 & 1-5 years<br>• **Quality Improvement Learning Collaborative (QILC),** a performance measure to improve screening and counseling on SDH and injury prevention	10 pediatric practices recruited from the AAP Ohio Chapter, AAP database<br>Cincinnati, Ohio
Ornelas et al. 2021 [74]	Mixed Methods Study (Literature Review, Interviews, and Root Cause Analysis)	Use of literature review and interviews of providers and patients; use of thematic analysis, to identify underlying causes of health inequity in hospital readmission rates; information from literature search and interviews were integrated in root cause analysis to determine and recommend the elements of a more equitable HF care model that included: (1) SDH screening; (2) technological innovation; (3) optimization of space;(4) implicit bias training	ZSFG Hospital cardiology clinic– safety net hospital (provides health care regardless of insurance status or ability to pay) with a high prevalence of HF among its patients<br>San Francisco, California<br>Urban communities
Swamy et al. 2020 [51]	Quantitative Study (Cross-Sectional Survey)	Use of a Likert-based cross-sectional survey administered via Survey Monkey to elicit perceptions on SDH themes from caregivers, pediatric residents, and staff/faculty; use of the following SDH screening tools:<br>• **SEEK** to reduce child abuse by addressing SDH for age 0-5<br>• **WE CARE** for age 2-10	Pasadena-Pediatric and Adolescent Health Center (PA-PAHC)<br>Southeast Houston<br>Urban communities
Okafor et al. 2020 [75]	Mixed Methods Study (Hunger Vital Sign (HVS) Survey with Focus Group Interviews)	Use of 2-item **HVS** tool to assess food insecurity, using paper and electronic formats, completed by parents/guardians of patients below 18; survey read out in English or Spanish by medical assistant for patients with low literacy, or by translator if patients did not	2 FQHCs<br>1 pediatric clinic, 1 family medicine clinic, New Haven Connecticut<br>Urban-suburban areas

		speak either language fluently; use of focus group among pediatricians to identify the challenges and recommendations of universal screening in clinical settings	
Morris et al. 2020 [68]	Qualitative Study (Protocol Development & Pilot Feasibility Study)	Use of multi-phase protocol for ED-based SDH screening and referral for malnutrition and food insecurity among older adults and how to incorporate in existing clinic workflow as part of the BRIDGE Study; use of 2 screening tools:<br>• **2-item validated Malnutrition Screening Tool (MST)** recommended for older adults and already used in in-patient, acute care settings at UNC<br>• **2-item HVS** tool on food insecurity and recommended by AARP for older adults	FQHCs, 1 private pediatric clinic, 1
Kulkarni et al. 2023 [81]	Commentary/Perspective	Mentioned use of ICD-10 Z codes to categorize SDH-related services and SDH screening tools:<br>• **AHC-HRSN Tool, 2017** from CMS<br>• **PRAPARE** from the National Association of Community Health Centers<br>• **Health Leads Social Needs Screening Toolkit, 2018**	Nationwide clinics, outpatient programs, clinics, psychiatric treatment facilities, academic medical centers' population health initiatives<br>Urban/rural classification not mentioned<br>Others: Medical center, telehealth, care coordination, outreach programs, medical home model, informal social support counseling, referral mechanisms for federal feeding programs such as food banks, SNAP, Child & Adult Care Food Program, National School & Breakfast Programs
Berry et al. 2020 [69]	Qualitative Study	Use of **CMS ACH-HRSCN** screening tool and referral program in healthcare systems serving low-income patients; identifying lessons learned from implementation and feasibility of self-reported SDH screenings; CMS AHC screening tool was standardized and integrated in EHRs across all system facilities; results of social needs screenings were made available on patient charts using NowPow, a closed-loop electronic referral system	New York City Health + Hospitals (NYC H+H), largest US public health care system, and affiliated 3 ambulatory care clinics (adult outpatient and pediatric clinics) in Manhattan, and adult outpatient clinic in the South Bronx, New York<br>Urban communities
Brewster et al. 2020 [52]	Quantitative Study (Multivariate Regression)	Use of 2017-2018 **NSHOS** survey (n = 2178) to determine: (1) number of social risks systematically screened among patients; (2) extent of participation in value-based payment models; (3) capacity of physician practices for innovation; multivariate regression models were used to examine predictors of social risk screening	2178 physician practices<br>National sample
Cordova-Ramos et al. 2022 [76]	Mixed Methods Study	Use of an electronic survey among clinical leaders in a random sample of 100 hospitals with level 2 to 4 NICUs to determine national prevalence, predictors, and characteristics of standardized SDH screenings, and provider beliefs on SDH screening in the NICU setting; mentioned potential use of institution-developed SDH screening tool or any of the following: **AHC HRSN Tool; iHELP Social History Tool; PRAPARE; SEEK;** and **WE CARE**	100 hospitals with level 2-4 NICUs<br>Located 5 US regions
Horwitz et al. 2020 [77]	Mixed Methods Study (Literature Review with Quantitative Analysis)	Use of LexisNexis and Google to search for articles and press releases to assess scope and scale of investments in upstream social determinants made by health systems; used Chi-Square and t-tests for categorical and continuous variables	626 healthcare systems involving 917 hospitals<br>National sample
Selvaraj et al. 2019 [53]	Quantitative Study	Development of a 13-item universal screening tool, "**Addressing Social Key Questions for Health Questionnaire (ASK)**" to identify social factors associated with ACEs; impact of screening on referral rates; and the feasibility and acceptability of screening in a medical home setting	4 academic pediatric primary care clinics, Chicago, Illinois<br>Urban communities
Meyer et al. 2020 [54]	Quantitative Study (Feasibility Study)	Development, implementation, and feasibility testing of own universal SDH screening that was incorporated in EMRs to identify unmet	Academic medical center - New York Presbyterian Hospital and 4 hospital-affiliated, community-based, out-

		patient social needs that would otherwise go undiscovered; study formed multidisciplinary teams (physician champions and practice administrators) who implemented SDH screening in waiting rooms without jeopardizing clinical workflow	patient primary care practices: Pediatrics, Internal Medicine, OB-GYN, Northern Manhattan, New York Urban communities
Strenth et al. 2022 [55]	Quantitative Study (Cross-Sectional Survey with Mediation Analysis)	Use of data from a cross-sectional survey administered to a practiced-based research network (PBRN) of family medicine residency programs to determine the influence of SDH and interpersonal violence on the management of type 2 diabetes; use of the following screening tools: 10-item **ACE Survey** by Felitti et al.; 4-item **HITS** screening tool on interpersonal violence; and 17-item **DDS** scale on diabetes-related distress; with patients' most recent HbA1c level as the primary outcome	7 Family Medicine residency programs in a practiced-based research network (PBRN), Texas
Tung et al. 2022 [56]	Quantitative Study (Multi-Site Cross-Sectional Survey)	Use of data from a multi-site cross-sectional study conducted by SIREN; utilized modified **Berkman-Syme Social Network Index** for isolation screening and assistance in primary care settings and to determine if screening discomfort and desire for assistance were associated with severity of social isolation; use of **NASEM** 1-item tool for financial strain; 4-item **HARK** tool for intimate partner violence; study mentioned other SDH screening tools: **NASEM** recommended measures, **PRAPARE**, and **CMS AHC** tool.	3 Academic primary care clinics in Boston, Chicago, and San Francisco Urban communities
Gruß et al. 2021 [70]	Qualitative Study (Formative Substudy of ASCEND)	Use of interviews with CHC staff to determine how CHCs introduced and integrated EHR-based SDH screening in clinic workflows and identified factors that affected the adoption of SDH screening	8 safety net CHCs in 5 US states: Oregon (8 CHCs); California (4 CHCs); Minnesota (1 CHC); Indiana (1 CHC): North Carolina (1 CHC)
Khatib et al. 2022 [57]	Quantitative Study (Retrospective Cross-Sectional Analysis)	Use of a retrospective cross-sectional analysis of EHRs to test **NowPow**, a digital platform, for screening and referring patients for social needs based on a modified **PRAPARE**; EHRs were linked to referral data to describe patient clinical and demographic characteristics; CHWs asked screening questions on social needs like food, housing, transportation	3 community hospitals South Chicago, Chicago, Illinois
Avallone et al. 2020 [71]	Qualitative Study (Feasibility Study on the Educational Training of Nursing Students)	Use of IHI 4Ms framework on age-friendly health systems and communities: "Matters, Mobility, Medications, and Mentation," in training 15 senior nursing students in caring for seniors and elderly who were living in a high-rise apartment building; students worked with an interprofessional team of bilingual social workers	Academic-practice partnership: Rutgers School of Nursing, Camden, New Jersey Northgate II, 23-story high-rise building with 308 apartments Urban community with 41% of residents at or below 100% federal poverty level
Montez et al. 2021 [58]	Quantitative Study (Retrospective Cohort Study with Bivariate Analysis and Mixed Effects Logistic Regression)	Use of 2-item **HVS** paper-based survey, in English and Spanish, to identify food insecurity trends over a two-year period among children aged 0–18 years with their parents/guardians having been screened at least once a year during the two-year period; patient demographic data were obtained from EHRs	Academic primary care clinic with a pediatric residency program and continuity clinics, Forsyth County, North Carolina, serving low-income, urban, and Medicaid-insured population
Morone 2017 [82]	Literature Review	Use of a review of literature from 3 databases: PsychInfo, CINAHL and PubMed to identify and evaluate available pediatric SDH screening tools	Primary care pediatric clinics US-based studies
Miller et al. 2022 [59]	Quantitative Study (Cross-Sectional Cohort)	Use of an osteoporosis risk assessment tool by BHT via clinical dashboard to identify, screen, and treat rural veterans at risk for osteoporosis through phone and US Postal Service communications; clinical dashboard was populated with data from the VHA Corporate Data Warehouse; no specific SDH screening tool mentioned	Mountain West region, US Rural communities

Fraze et al. 2021 [72]	Qualitative Study (Semi-Structured Interviews)	Use of semi-structured interviews with leaders and frontline staff from 29 health care organizations on how case management-based programs were developed and implemented for addressing and referring patient-level SDH needs to community-based organizations	29 different health care organizations from small town to multi-state health systems in regional locations in the US: South (4), Midwest (5); West (9); and Northeast (10)
Power-Hays et al. 2020 [60]	Quantitative Study	Use of EMRs of pediatric patients with SCD to collect data on household material hardship and their association with ED visits	Boston Medical Center Network, Boston, Massachusetts<br>Urban communities
Carter and Mazzoni 2021 [83]	Clinical Opinion	Theoretical discussion on the benefits of group prenatal care as a "viable vehicle" for addressing perinatal inequities to: (1) improve pregnancy outcomes in Black women; (2) increase quantity and quality of patient-practitioner interactions; (3) increase cross-cultural exposure to reduce clinician implicit and explicit bias and racism	Safety net clinics, FQHCs, academic medical centers, group prenatal care<br>St. Louis, Missouri
Gerend and Pai 2008 [84]	Literature Review	Use of a literature review using MEDLINE, CANCERLIT, and HealthSTAR databases on the social, economic, and cultural factors that may contribute to Black-White disparities in breast cancer mortality	Primary care clinics<br>National Health Interview Survey (NHIS)<br>US-based studies
Roland et al. 2017 [79]	Systematic Review	Use of a systematic review of 5 databases on studies on cancer-related CHW/patient navigator-based interventions in partnerships with FQHCs, in which majority was on breast, cervical, or colorectal cancer screenings and/or referrals	FQHCs, CHCs<br>US-based studies
Hamilton et al. 2022 [61]	Quantitative Study (Retrospective Observational Study with Cox Regression Analysis)	Use of an observational study to identify the association between census tract-level socioeconomic and neighborhood factors and prolonged PICU stay (>18 months) for children with severe sepsis; used mixed effects Cox regression models to determine the association of each socioeconomic factor with length of hospital stay	PICU<br>Shelby County, Tennessee
Tully et al. 2022 [73]	Qualitative Study (Interviews & Focus Groups)	Use of individual interviews (n = 15), focus group (n = 1) with 11 maternity health care team members, and a focus group of Spanish-speaking patients (n = 4) to identify the perspectives and concerns of patients and health care team on SDH screening in maternity care	Prenatal clinic of an academic medical center that participates in the Medicaid Pregnancy Medical Home Program<br>Southeastern United States
Fort et al. 2022 [62]	Quantitative Study (Case-Control Study)	Use of a retrospective chart review to identify patients with food insecurity and to determine consistency in food documentation in medical charts; bivariate analyses used to describe demographic, clinical, and health care utilization characteristics; positive screens were given food bags from a local food bank and sticker placed on patients' paper registry	Kaiser Permanente pediatric clinic<br>Oakland, California
Chukmaitov et al. 2022 [63]	Quantitative Study (Cross-sectional Study)	Use of a pilot study to screen and address for social risk factors among in-patients in an academic medical center; screening tool was patterned after the **Health Leads Social Needs Screening**; SDH data and EHRs were linked to determine associations between food, housing, transportation, and demographic factors with clinical characteristics to inform discharge plans and link patients to community resources	VCU Medical Center – urban safety net academic medical center, Internal Medicine Unit<br>Richmond, Virginia
Hughes 2016 [85]	Commentary	Cited use of various strategies to assess SDH issues in clinical settings:<br>• **ACE survey** in rural practices<br>• **WellRX** pilot on housing, income, food insecurity, substance abuse, transportation, and employment<br>• 14-item **SDH survey** using electronic tablets<br>• SDH screening by family physicians and to intervene as appropriate<br>• Conduct SDH research, data collection, and analysis in primary care settings to inform	Doctor's offices, FQHCs<br>Nationwide

		patient care, population health, and policy interventions	
Quiñones and Hammad 2020 [86]	Literature Review	Use of a literature review to determine impact of SDH on the onset and progression of CKD and ESRD in primary care settings; SDH screening strategies and tools mentioned: (1) machine learning tools to identify patients at-risk of adverse SDH from available data; (2) implementing EHR-incorporated SDH screening in an urban safety net primary care setting;(3) use of **CLEAR** electronic SDH toolkit, **CMS AHC**, and **AAP Neighborhood Navigator**	Primary care settings
Webb 2020 [87]	Literature Review	Use of literature review and clinical cases to illustrate the impact of SDH on a chronically-transfused population with SCD; determine role of health literacy in complex medical decision-making and communication strategies; identify health-related quality of life measures; article discussed 7 SDH screening tools: **SEEK**, **HealthBegins Upstream Risks Screening Tool, CMS AHC HRSN Tool, iScreen, Addressing Social Key Questions for Health (ASK), WE CARE,** and **Family Needs Screening Program**	Hospital settings<br>Locations not specified
Kim-Mozeleski et al. 2022 [64]	Quantitative Study (Descriptive and Logistic Regression Analyses)	Use of EHRs to analyze the association between social needs (food insecurity, financial strain, transportation barriers, housing/utility insecurity) and smoking status among adult patients	MetroHealth System, Cleveland, Ohio<br>MetroHealth System - safety-net health care system (4 hospitals, 4 EDs, >20 health centers in the county), Cleveland, Cuyahoga County, Ohio
Massar et al. 2022 [78]	Mixed Methods Study (Process Measures and In-Depth Interviews)	Use of 48 in-depth qualitative interviews with leadership, providers and staff as part of a multiple case study of social needs screening and referral programs	4 pediatric primary care clinics housed in diverse settings (FQHC, academic hospital, small community hospital, large non-profit health system), New York City, New York

Table Abbreviations: AAFP-American Academy of Family Physicians; AAP-American Academy of Pediatrics; AARP-American Association of Retired Persons; ACA-Patient Protection and Affordable Care Act; ACEs-Adverse Childhood Experiences; AHC-HRSN-Accountable Health Communities Health-Related Social Needs Screening; ASCEND Study-Approaches to Community Health Center Implementation of Social Determinants of Health Data Collection and Action; ASK Questionnaire-Addressing Social Key Questions for Health Questionnaire; BHT-Bone Health Team; BRFSS-Behavior Risk Factor Surveillance System; BRIDGE-Building Resilience and InDependence for Geriatric Patients in the Emergency Department; CAD-Coronary Artery Disease; CEOs-Chief Executive Officers; CHCs-Community Health Centers; CHD-Coronary Heart Disease; CHF-Congestive Heart Failure; CHNAs-Community Health Needs Assessments; CHWs-Community Health Workers; CINAHL-Cumulative Index to Nursing and Allied Health Literature; CKD-Chronic Kidney Disease; CLEAR Toolkit-Community Leadership on the Environment, Advocacy, and Resilience; CMS-Centers for Medicare and Medicaid Services; COPD-Chronic Obstructive Pulmonary Disease; CVD-Cardiovascular Disease; DDS-Diabetes Distress Scale; ED-Emergency Department; EHRs-Electronic Health Records; EMRs-Electronic Medical Records; ESRD-End-Stage Renal Disease; FQHCs-Federally-Qualified Health Centers; HARK Tool-Humiliation, Afraid, Rape, Kick; HITS Tool-Hurt-Insult-Threaten-Scream; HIV-Human Immuno-deficiency Virus; HVS-Hunger Vital Sign; ICD-International Classification of Diseases; ICU-Intensive Care Unit; iHELP-Income/Insurance-Hunger/Housing Conditions/Homeless-Education/Ensuring Safety-Legal Status, Literacy-Personal Safety; IHI-Institute for Healthcare Improvement; IRS-Internal Revenue Service; MI-Myocardial Infarction; NASEM-National Academies of Sciences, Engineering, and Medicine; NHANES-National Health and Nutrition Examination Survey; NHIS-National Health Interview Survey; NLCHCs-Nurse-Led Community Health Centers; NICU-Neonatal Intensive Care Unit; NSHOS-National Survey of Healthcare Organizations and Systems; OB-GYN-Obstetrics-Gynecology; PDSA-Plan-Do-Study-Act; PICU-Pediatric Intensive Care Unit; PHQ-9-Patient Health Questionnaire-9; PRAPARE-Protocol for Responding to and Assessing Patient Assets, Risks, and Experiences; PSA-Prostate Specific Antigen; QI-Quality Improvement; SCD-Sickle Cell Disease; SDH-Social Determinants of Health; SEEK-Safe Environment for Every Kid; SIREN- Social Interventions Research & Evaluation Network; SNAP-Supplemental Nutrition Assistance Program; VCU-Virginia Commonwealth University; VHA-Veterans Health Administration; WE CARE-Well Childcare Visit, Evaluation, Community Resources, Advocacy, Referral, Education; ZSFG-Zuckerberg San Francisco General
